# Preliminary assessment of TNM classification performance for pancreatic cancer in Japanese radiology reports using GPT-4

**DOI:** 10.1007/s11604-024-01643-y

**Published:** 2024-08-20

**Authors:** Kazufumi Suzuki, Hiroki Yamada, Hiroshi Yamazaki, Goro Honda, Shuji Sakai

**Affiliations:** 1 Department of Radiology, Division of Diagnostic Imaging and Nuclear Medicine, Tokyo Women’s Medical University, 8-1, Kawada-Cho, Shinjuku-Ku, Tokyo 162-8666 Japan; 2 Department of Radiology, Iwaki City Medical Center, Iwaki City, Japan; 3 Department of Surgery, Division of Hepatobiliary and Pancreatic Surgery, Tokyo Women’s Medical University, Tokyo, Japan

**Keywords:** GPT-4, Large-scale language model, Natural language processing, Pancreatic cancer, TNM classification

## Abstract

**Purpose:**

A large-scale language model is expected to have been trained with a large volume of data including cancer treatment protocols. The current study aimed to investigate the use of generative pretrained transformer 4 (GPT-4) for identifying the TNM classification of pancreatic cancers from existing radiology reports written in Japanese.

**Materials and methods:**

We screened 100 consecutive radiology reports on computed tomography scan for pancreatic cancer from April 2020 to June 2022. GPT-4 was requested to classify the TNM from the radiology reports based on the General Rules for the Study of Pancreatic Cancer 7th Edition. The accuracy and kappa coefficient of the TNM classifications by GPT-4 was evaluated with the classifications by two experienced abdominal radiologists as gold standard.

**Results:**

The accuracy values of the *T*, *N*, and *M* factors were 0.73, 0.91, and 0.93, respectively. The kappa coefficients were 0.45 for *T*, 0.79 for *N*, and 0.83 for *M*.

**Conclusion:**

Although GPT is familiar with the TNM classification for pancreatic cancer, its performance in classifying actual cases in this experiment may not be adequate.

**Supplementary Information:**

The online version contains supplementary material available at 10.1007/s11604-024-01643-y.

## Introduction

Advancements in the natural language processing technology have facilitated the categorizing and summarizing of radiology reports [1–5]. Large-scale language models (LLMs), such as generative pretrained transformer 4 (GPT-4) (OpenAI Inc., San Francisco, CA, USA) are anticipated to have been trained on a large amount of various medical resources and tested for many medical applications, including interpretation of medical documents and radiology reports [6–8]. One of these applications is determining cancer staging from the natural language text of radiology reports without the need for additional training [9, 10].

The current study aimed to investigate the use of GPT-4 for accurately classifying the TNM stage of pancreatic cancer based on unstructured radiology reports written in Japanese. Previous studies have applied LLMs to the TNM classification of lung cancer [9, 10]. This study is the first to investigate the TNM classification of pancreatic cancer using GPT-4.

## Materials and methods

### Patients and ethics approval

We screened 100 consecutive patients with pancreatic cancer (including those suspected of cancer) who underwent contrast-enhanced abdominal computed tomography (CT) scan and evaluation of TNM classification on radiology report starting from April 2020 at our institution and who had available radiology report on TNM classification. In cases requiring a second TNM classification during the study period, such as after neoadjuvant chemotherapy, the later report was excluded, and the collection continued until 100 cases were reached. No additional exclusion criteria were applied. These radiology reports were created by modifying shared text templates in most cases; thus, they were written in a similar style. Moreover, pancreatic cancer was selected as the number of cases and radiology reports available at our institution were sufficient based on the clinical situation at our hospital. Furthermore, our team included abdominal radiology specialists.

This study was approved by the Institutional Review Board with a waiver for documented informed consent from patients (approval number 2023–0029). The research title was published on an institutional website, and the patients were allowed to opt-out based on the institutional regulations. Patient identifiers were removed, and this study was conducted in accordance with the Declaration of Helsinki. This was a retrospective study, and we have reviewed our study to eliminate bias according to the Standards for Reporting of Diagnostic Accuracy (STARD) guidelines. Although our sample of 100 consecutive pancreatic cancer cases may not be sufficient to adhere to the guidelines, we have ensured that other criteria are met in our study.

### Data processing

Our radiology reports provide an objective description of image findings and impressions from a radiologist’s perspective based on those findings. The TNM classification of cancer is often included by the impressions as the result of interpretations; however, we did not use this original TNM classification in this study. Image findings of 100 cases were reviewed by two experienced abdominal radiologists (with 21 years and 13 years of experience as radiologists) to determine the TNM classification according to the General Rules for the Study of Pancreatic Cancer 7th Edition [11]. In cases where there was a disagreement between the two radiologists, a consensus was reached through discussion to determine the gold standard (TNM by radiologists). Only the descripted image findings were transmitted to GPT-4 to identify the TNM classification (TNM by GPT-4). No personally identifiable data, metadata, such as age and gender, or TNM classifications made by radiologists were transmitted to GPT-4. OpenAI Playground was used to prevent the use of our data used for training by GPT. The latest language model “gpt-4-turbo-2024–04-09” was utilized. The temperature parameter, which controls variability, was set to 0.0. The system prompt was designed to guide GPT-4 in classifying the TNM stage based on the radiology reports and the General Rules for the Study of Pancreatic Cancer 7th Edition. Our prompt uses the chain-of-thought technique as prompt engineering [12]. The prompt tells GPT-4 to determine the *T*, *N*, and *M* factors step-by-step and then summarizes them into the TxNxMx format.

Prompt for GPT-4 (Translation into English, as original prompt was written in Japanese):

“*Identify TNM classification from the CT scan findings according to the General Rules for the Study of Pancreatic Cancer 7th Edition.*

- *Evaluate the T factor based on the tumor size, local extent of pancreatic cancer (CH, DU, S, RP, PV, A, PL, and OO), and the description about invasion into the surrounding areas.*
- *Evaluate the N factor based on the presence or absence of metastasis to the regional lymph nodes.*
- *Evaluate the M factor based on the presence or absence of distant metastasis.*

*Finally, summarize them in the TxNxMx format.*”

### Evaluation

The performance of TNM by GPT-4 was assessed using the accuracy and kappa coefficient of the *T*, *N*, and *M* factors versus the TNM by radiologists. Microsoft Excel (Microsoft, Redmond, WA, USA) and JMP Pro 17 (SAS Institute, Cary, NC, USA) were used to calculate these indices.

For T1a and T1b classifications by GPT-4, they were considered correct if they corresponded to T1 by the radiologists. If the radiologists marked the cases as Nx or Mx, indicating no clear documentation of metastasis, any GPT-4’s classification of N and M factors was considered correct. Radiologists reserved judgment on the presence of lymph node metastasis in such cases, including lymph node around aorta.

## Results

In total, 100 consecutive patients were identified from April 2020 to June 2022. Among them, 62 were men and 38 women and were aged 38–93 (average: 70.3 ± 10.8) years. The TNM classifications made by radiologists for all patients were as follows: T1 (*n* = 5), T2 (*n* = 2), T3 (*n* = 64), T4 (*n* = 28), and Tx (*n* = 1). For the *N* factor, N0 (n = 68), N1 (n = 16), and Nx (n = 16) were reported. For the *M* factor, M0 (*n* = 66), M1 (*n* = 29), and Mx (*n* = 5) were observed. The classification made by GPT-4 were as follows: T1a (*n* = 2), T1b (*n* = 2), T2 (*n* = 1), T3 (*n* = 66), and T4 (*n* = 29). GPT-4 reported N0 (*n* = 65) and N1 (*n* = 35) for the *N* factor and M0 (*n* = 74) and M1 (*n* = 26) for the *M* factor (Tables 1, 2, and 3).

**Table 1:** Classification of *T* factor by radiologists and GPT-4

	T1 by radiologist	T2 by radiologist	T3 by radiologist	T4 by radiologist	Tx by radiologist
T1a by GPT-4	2	0	0	0	0
T1b by GPT-4	1	0	1	0	0
T2 by GPT-4	1	0	0	0	0
T3 by GPT-4	1	2	52	10	1
T4 by GPT-4	0	0	11	18	0

**Table 2:** Classification of *N* factor by radiologists and GPT-4

	N0 by radiologist	N1 by radiologist	Nx by radiologist
N0 by GPT-4	59	0	6
N1 by GPT-4	9	16	10

**Table 3:** Classification of *M* factor by radiologists and GPT-4

	M0 by radiologist	M1 by radiologist	Mx by radiologist
M0 by GPT-4	65	6	3
M1 by GPT-4	1	23	2

The accuracy values were 0.73 for *T* (95% confidence interval [CI] 0.64–0.82), 0.91 for *N* (CI 0.85–0.97), and 0.93 (CI 0.88–0.98), respectively. The kappa coefficients were 0.45 for *T* (CI 0.28–0.62), 0.79 for *N* (CI 0.66–0.92), and 0.83 for *M* (CI 0.71–0.95).

## Discussion

The TNM classification is a crucial factor in determining treatment strategies, and it is expected that the classification provided by GPT will closely align with that provided by radiologists. However, the results of our experiment showed that the kappa values did not meet clinical standards. The errors have various causes, making it difficult to identify all of them. Therefore, we focused on identifying common conditions that seemed to be involved in the majority of error cases.

The *T* factor in the General Rules for the Study of Pancreatic Cancer 7th Edition aligns with the 7th edition of the TNM Classification of Malignant Tumors by the Union for International Cancer Control (UICC classification). In these guidelines, T4 is defined as invasion (including “abutment” and “contact”) into the celiac axis or superior mesenteric artery. In nine cases, GPT-4 did not classify “contact” with these arteries as invasion, incorrectly determining them as T3. In 11 cases, GPT-4 incorrectly classified invasions into other organs, such as the spleen, and invasions into the portal vein or splenic artery as T4. The extent of invasion is represented by symbols, such as CH, DU, S, RP, PV, A, PL, and OO, and GPT-4 has a high accuracy in recognizing symbols and notations representing the extent of invasion. We guided GPT-4 to interpret these symbols; however, their meaning was not explained in the prompt. GPT-4 interpreted these symbols based on its own knowledge.

The low accuracy in *N* and *M* factor classification was attributed to the different categories of metastases to the lymph nodes outside the regional lymph nodes. Under the General Rules for the Study of Pancreatic Cancer 7th Edition, metastasis to nonregional lymph nodes, such as the para-aortic lymph nodes, is considered distant metastasis and is classified as M1. However, GPT-4 incorrectly classified it as N1 in six cases. There is a concern that such classification errors could occur with other types of cancer as well.

Another reason might be that Japanese radiology reports are rarely published; thus, GPT-4 may not have sufficiently learned their descriptive expressions. Furthermore, discrepancies between the evaluations by two radiologists were noted in seven cases for the *T* factor, eight for the *N* factor, and two for the *M* factor, and GPT-4 failed most of these subtle cases, correctly identifying only one case each for the *T*, *N*, and *M* factors. This indicates that Japanese language expressions are often ambiguous, resulting in varying interpretations even among radiologists.

LLMs including GPT-4 do not retain memory unlike a database. Their knowledge is fragmented and diffused within the mass of neural network parameters, resulting in a state similar to a vague recollection. Therefore, even though the guidelines were explicitly named, GPT-4 defaulted to a more general manner of classifying. For example, direct invasion to other organs is classified as T4, and lymph node metastases as N1. When asked how pancreatic cancer is classified as T4, GPT-4 can correctly respond that it involves invasion into the celiac axis or the superior mesenteric artery. This phenomenon likely occurred due to insufficient training on the guidelines. This issue persists even when specifying the UICC classification instead of the Japanese guidelines. If this issue is pointed during interactions, GPT-4 can optionally correct it according to the notification by users. Individual rules can be enforced for *N* and *M* factors in the prompt. However, such approach is not in accordance with our objective to develop an assistant for TNM classification for all types of cancers or a general-purpose assistant for imaging diagnosis.

The current study had several limitations. That is, the results were not compared with those of pathological examinations. Beyond this study, a hypothesis may exist, which states that LLMs might be able to interpret radiology reports more effectively than radiologists. However, in this study, we focused on the ability of LLMs as natural language processors with medical knowledge. Although the evaluation of only 100 cases in this study may be insufficient, significantly increasing the number of cases beyond this was difficult due to practical conditions. We attempted to eliminate selection bias by including consecutive cases; however, a distribution bias may still exist. This may be due to challenges in detecting early-stage pancreatic cancer and the tendency of our facility to receive advanced cases from neighboring facilities. That is, 64 patients presented with T3 tumors, and only five and two patients had T1 and T2 tumors, respectively. This bias could lead to an apparent increase in accuracy compared with those of previous reports about lung cancer [9, 10]. For better evaluation, the kappa coefficient is used as an alternate indicator that eliminates the effects of coincidental matches, and the kappa coefficient for the *T* factor remains low at 0.45 (moderate agreement). Since the radiologists reserved judgment on the presence of metastasis to slightly enlarged lymph nodes and used Nx or Mx, it was necessary to consider GPT-4’s responses to be correct. This improved the evaluation of the *N* and *M* factors slightly. Further research is needed, including evaluations with improved prompts, cancers of other organs, indicators beyond the TNM classification, and alternative LLMs besides GPT-4.

Another issue was the lack of reproducibility of GPT’s responses. OpenAI provides a parameter called “temperature” to control the variability of GPT’s responses, which can be specified as a decimal between 0 and 1. When using GPT as a chatbot, the temperature is often set to a value higher than 0.0 to make it behave more human-like. However, for scientific experiments requiring reproducibility, the temperature is commonly set to 0.0. Even with the temperature set to 0.0, the variability of GPT-4’s responses may not be sufficiently suppressed. Whether the variability in GPT-4’s responses affects TNM classification requires further investigation. In addition, OpenAI frequently updates the language model under GPT-4, which raises concerns about maintaining the reproducibility of experimental results.

In similar previous studies using lung cancer, the TNM classification was provided in the prompt [9, 10]; however, the prompt we used did not include the definitions of the TNM classification. Although the performance was insufficient, GPT can act as an artificial intelligence with medical knowledge. When the training of LLMs advances further, they will become useful copilots for radiologists.

## Conclusion

GPT is familiar with the TNM classification for pancreatic cancer; however, in this experiment, its performance in classifying actual cases may be inadequate. Further investigation, particularly, on how prompts are formulated is needed.

## Supplementary Information

Below is the link to the electronic supplementary material.

Supplementary file1 (PDF 229 KB)

Supplementary file2 (PDF 72 KB)
