# Supplementary material for: Preliminary assessment of TNM classification performance for pancreatic cancer in Japanese radiology reports using GPT-4
Source: Jpn J Radiol. 2024 Aug 20;43(1):51–5. doi: 10.1007/s11604-024-01643-y (PMC11717849; doi:10.1007/s11604-024-01643-y)
Supplement: Supplementary file 1 — Supplementary file1 (PDF 229 KB) [file 11604_2024_1643_MOESM1_ESM.pdf]

**User prompt (data sent to GPT-4), findings of radiological report:**

"膵体部に横断像で 22x14mm の腫瘤（内部不均一。乏血性。辺縁不整。膵癌として矛盾せず。）がある。

下記浸潤を疑う。

CH0, DU0, S1s/o, RP1s/o, PV0, AXsp, PL0, OO0

SMA/CA/CHA に接触なし、SPA とは一部わずかに接するか

SMV/PV に接触なし、SPV に近接するが不整像はなし

上流主膵管拡張と膵実質の萎縮がある。

胆管拡張がある。

胆道系拡張はない。

傍大動脈領域のリンパ節が目立つ。その他有意なリンパ節腫大はない。

腹水、播種を示唆する所見はない。

遠隔転移（肝、肺等を含む）はない。

肝嚢胞、腎嚢胞がある。

動脈、静脈、門脈系に有意な正常変異はない。

その他撮像範囲に有意な異常はない。"

**Assistant prompt (generated by GPT-4) including suggested TNM classification:**

このCT画像所見に基づいて、膵癌のTNM分類を以下のように決定します。
**T因子:**

- 腫瘍の大きさは22×14mmであり、膵体部に位置しています。
- 浸潤の疑いがある領域はS1s/o（脾動脈への浸潤の疑い）、RP1s/o（後腹膜への浸潤の疑い）、AXsp（腹腔動脈周囲への浸潤の疑い）です。
- SMA/CA/CHA（上腸間膜動脈、腹腔動脈、肝動脈）に接触はなく、SPA（脾動脈）とはわずかに接するかもしれませんが、SMV/PV（上腸間膜静脈/門脈）には接触がなく、SPV（脾静脈）に近接していますが不整像はありません。
- これらの情報から、T3（脾動脈や上腸間膜動脈への浸潤が疑われるが、門脈や上腸間膜静脈への明確な浸潤はない）と評価します。

**N因子:**

- 傍大動脈領域のリンパ節が目立つとの記述がありますが、その他に有意なリンパ節腫大は記載されていません。
- この情報から、N1（リンパ節転移が疑われる）と評価します。

**M因子:**

- 遠隔転移は記載されておらず、肝嚢胞や腎嚢胞は遠隔転移とは関連しないため、M0（遠隔転移なし）と評価します。

最終的なTNM分類は **T3N1M0** です。

**Translation of assistant prompt in English:**

"*Based on the CT scan findings, the TNM classification of pancreatic cancer is determined as follows:*

***T factor:***

*The tumor size was 22 × 14 mm and was located in the pancreatic body.*

*The suspected areas of invasion include S1s/o (suspicion of splenic artery invasion), RP1s/o (suspicion of retroperitoneal invasion), and AXsp (suspicion of invasion around the celiac axis).*

*There was no contact with the superior mesenteric artery, celiac artery, and common hepatic artery (SMA/CA/CHA, respectively). There might be a slight contact with the splenic artery. However, there was no contact with the superior mesenteric vein/portal vein (SMV/PV), and it was close to the splenic vein (SPV) without any irregularity.*

*Based on this information, the tumor was classified as T3. Splenic artery invasion and superior mesenteric artery invasion were suspected. However, there was no clear invasion to the portal vein or superior mesenteric vein.*

***N factor:***

*The lymph nodes around the aorta were prominent. However, no other lymph nodes were significant enlarged. Based on this information, the tumor was classified as N1 (suspected lymph node metastasis).*

***M factor:***

*Distant metastasis was not observed, and liver or kidney cysts were not related to distant metastasis. However, the tumor was classified as M0 (no distant metastasis).*

*The final TNM classification was* ***T3N1M0****.*”

Within the reply of GPT, the absence of invasion to the superior mesenteric artery was mistakenly changed to presence. This might be referred to as hallucination of LLMs. GPT is likely influenced by patterns from similar texts that it has previously learned. In addition, metastasis to the para-aortic lymph nodes should be classified as M1. However, it was incorrectly identified as N1. Owing to the uncertainty of metastasis to the para-aortic lymph nodes, this study was tentatively classified by the radiologist as T3N0Mx.
