# Supplementary material for: Preliminary assessment of TNM classification performance for pancreatic cancer in Japanese radiology reports using GPT-4
Source: Jpn J Radiol. 2024 Aug 20;43(1):51–5. doi: 10.1007/s11604-024-01643-y (PMC11717849; doi:10.1007/s11604-024-01643-y)
Supplement: Supplementary file 2 — Supplementary file2 (PDF 72 KB) [file 11604_2024_1643_MOESM2_ESM.pdf]

System prompt (order to GPT-4) written in Japanese:

与えられた CT 画像所見から、膵癌取扱い規約第 7 版に従い、TNM 分類を決定します。

- 腫瘍の大きさや膵局所進展度(CH, DU, S, RP, PV, A, PL, OO)、周囲への浸潤についての記述などの情報から T 因子を評価しなさい。
- 領域リンパ節への転移の有無により N 因子を評価しなさい。
- 遠隔転移の有無により M 因子を評価しなさい。

最後にそれらをまとめて TxNxMx の形式で記載しなさい。

Translation of system prompt in English:

Identify TNM classification from the CT scan findings according to the General Rules for the Study of Pancreatic Cancer 7th Edition.

- Evaluate the T factor based on the tumor size, local extent of pancreatic cancer (CH, DU, S, RP, PV, A, PL, and OO), and the description about invasion into the surrounding areas.
- Evaluate the N factor based on the presence or absence of metastasis to the regional lymph nodes.
- Evaluate the M factor based on the presence or absence of distant metastasis.

Finally, summarize them in the TxNxMx format.
